# Stabilization of Brain Mast Cells Alleviates LPS-Induced Neuroinflammation by Inhibiting Microglia Activation

**DOI:** 10.3389/fncel.2019.00191

**Published:** 2019-05-03

**Authors:** Hongquan Dong, Yiming Wang, Xiaojun Zhang, Xiang Zhang, Yanning Qian, Haixia Ding, Shu Zhang

**Affiliations:** ^1^Clinical Research Center, The First Affiliated Hospital of Nanjing Medical University, Nanjing, China; ^2^Department of Anesthesiology, The First Affiliated Hospital of Nanjing Medical University, Nanjing, China; ^3^Infection and Immunity Theme, South Australian Health and Medical Research Institute, Adelaide, SA, Australia; ^4^School of Medicine, College of Medicine and Public Health, Flinders University, Bedford Park, SA, Australia; ^5^Department of Rheumatology, The First Affiliated Hospital of Nanjing Medical University, Nanjing, China; ^6^Department of Anesthesiology, Shanghai First People’s Hospital, Shanghai, China; ^7^Department of Geriatric, The First Affiliated Hospital of Nanjing Medical University, Nanjing, China

**Keywords:** mast cells, LPS, microglia activation, neuroinflammation, migtation

## Abstract

**Background:**

The functional aspects of mast cell-microglia interactions are important in neuroinflammation. Our previous studies have demonstrated that mast cell degranulation can directly induce microglia activation. However, the role of mast cells in Lipopolysaccharide (LPS)-induced microglia activation, neuroinflammation and cognitive impairment has not been clarified.

**Methods:**

This study investigated the interaction between brain microglia and mast cells *in vivo* through site-directed injection of cromolyn into rat right hypothalamus using stereotaxic techniques. Cognitive function was subsequently assessed using trace fear conditioning and Y maze tests. Mast cells in rat brain were stained with toluidine blue and counted using Cell D software. Microglia activation was assessed by Iba1 immunohistochemistry both in rat brain and in mast cell-deficient Kit^W-sh/W-sh^ mice. Receptor expression in rat microglia was determined using flow cytometry analysis. Cytokine levels in rat brain tissue and cell supernatant were measured using high-throughput ELISA. Western blotting was used to analyze Cell signaling proteins.

**Results:**

In this study, intraperitoneal injection of 1 mg/kg LPS induced mast cell activation in hypothalamus and cognitive dysfunction in rats, and that this process can be repressed by the mast cell stabilizer cromolyn (200 μg). Meanwhile, in mice, LPS IP injection induced significant microglia activation 24 h later in the hypothalamus of wild-type (WT) mice, but had little effect in Kit^W-sh/W-sh^ mice. The stabilization of mast cells in rats inhibited LPS-induced microglia activation, inflammatory factors release, and the activation of MAPK, AKT, and NF-κB signaling pathways. We also found that LPS selectively provokes upregulation of H_1_R, H_4_R, PAR2, and TLR4, but downregulation of H_2_R and H_3_R, in ipsilateral hypothalamus microglia; these effects were partially inhibited by cromolyn. In addition, LPS was also found to induce activation of P815 cells *in vitro*, consistent with findings from *in vivo* experiments. These activated P815 cells also induced cytokine release from microglia, which was mediated by the MAPK signaling pathway.

**Conclusion:**

Taken together, our results demonstrate that stabilization of mast cells can inhibit LPS-induced neuroinflammation and memory impairment, suggesting a novel treatment strategy for neuroinflammation-related diseases.

## Introduction

Neuroinflammation has been recognized as the chief culprit in multiple neurodegenerative diseases. An increasing number of studies suggest that communications between microglia, immune cells, and neurons might promote the acceleration of neuroinflammation and the exacerbation of neurodegenerative disorders (Block and Hong, 2005). However, the association between microglia and immune cells still remains to be fully explored.

Emerging evidence indicates that prolonged inflammatory responses involving astrocytes and microglia promote neurodegenerative disease exacerbation (Block and Hong, 2005). Microglia are the resident immune cells in central nervous system (CNS), providing immune surveillance. Under physiological conditions, microglia exhibit a resting state associated with the secretion of neurotrophic and anti-inflammatory factors (Streit, 2002). In pathological situations, microglia switch to an activated phenotype that initiates an inflammatory response. In most cases, this response is temporary and has beneficial effects in eradicating injured CNS cells. However, prolonged and inappropriate activation of microglia can lead to brain injury and neuronal apoptosis (Dauer and Przedborski, 2003). Hence, modulation and inhibition of the over-activation of microglia may provide a novel therapeutic target to improve treatment of neurodegenerative diseases.

Mast cells, notorious for their role in allergic diseases, reside close to microglia and neurons in the CNS, mainly present in the leptomeninges, blood vessels, thalamus and hypothalamus (Florenzano and Bentivoglio, 2000; Khalil et al., 2007). Mast cells store numerous proinflammatory mediators, such as histamine and proteases, in secretory granules, and can secrete them upon activation (Schwartz, 1987; Serafin and Austen, 1987; Galli, 1993; Dvorak, 1997; Galli et al., 2005). Notably, as the “first responder” cells of the CNS, mast cells can store preformed TNF-a in the secretory granules (Zhang B. et al., 2012). They can induce the secretion of pro-inflammatory cytokines from activated microglia via signaling through the H_1_R, H_4_R and PAR2 receptors (Zhang S. et al., 2012; Dong et al., 2014). As mast cells participate in the opening of the blood-brain barriers (BBB) and in neuroinflammation, meningeal mast cells are able to recruit an early wave of neutrophils and T cells to the CNS, leading to further inflammatory cell influx and exaggerated neuroinflammation (Sayed et al., 2010; Zhang S. et al., 2016). Owing to this pivotal role of mast cells in the pathobiology of neuroinflammation, it is important to determine the exact regulatory effects of activated mast cells on microglia in neuroinflammation.

We have previously reported that mast cell degranulation could directly induce microglia activation (Dong et al., 2017). However, whether mast cells affect LPS-induced microglia activation has not been reported. In the present study, we demonstrate that mast cell degranulation can aggravate LPS-induced neuroinflammation by evoking microglia activation. In addition, we also find that stabilization of mast cells can inhibit LPS-induced neuroinflammation and memory impairment.

## Materials and Methods

### Animals

Eighty-four adult male SD rats (each weighing about 250 g), 12 adult mast cell-deficient Kit^W-sh/W-sh^ mice and 12 littermate controls were used in this experiment, which were obtained from the Mode Animal Research Center of Nanjing University. All animals were housed under conditions previously described (Dong et al., 2017): five per cage, 50–60% humidity, 22°C constant room temperature and free access to food and water. All experiments were carried out according to the Guide for the Care and Use of Laboratory Animals of the National Institutes of Health (publication no. 85-23, revised 1985) and the Guidelines for the Care and Use of Animals in Neuroscience Research by the Society for Neuroscience. They were approved by IACUC (Institutional Animal Care and Use Committee of Nanjing Medical University, No: 14030126).

### Reagents

Dulbecco’s modified Eagle’s medium (DMEM) and fetal calf serum (FCS) were purchased from Gibco–BRL (Grand Island, NY, United States). Lipopolysaccharide (LPS, from Escherichia coli 0111:B4), disodium cromoglycate (cromolyn) were purchased from Sigma-Aldrich (St. Louis, MO, United States). Fluorescenc mounting medium with DAPI and anti-mast cell Tryptase antibody were purchased from Abcam (HK, China). Rabbit anti-Iba1 antibody was purchased from Wako Chemicals USA, Inc. Rabbit anti-H_1_R and anti-H_2_R antibodies were purchased from Alomone Labs (Jerusalem, Israel). Rabbit anti-H_3_R antibody was purchased from Abcam (Hong Kong, China). And rabbit anti-H_4_R was purchased from Santa Cruz (CA, United States). Fluorescein isothiocyanate (FITC) – conjugated mouse anti-OX-42 antibody and isotype control, phycoerythrin (PE)-conjugated goat anti-rabbit secondary antibody were purchased from BD (BD Biosciences, United States). Cytokines (TNF-α, IL-6, IL-1β, INF-γ, RANTES, and IL-10) measured kit was purchased from MERCK Millipore Corporation (Billerica, MA, United States). Specific rabbit anti-p38, anti-Phospho-p38, anti-JNK, anti-Phospho-JNK, anti-ERK, anti-Phospho-ERK, anti-AKT, anti-Phospho-AKT, and anti-Phospho-NF-κB p65 antibodies, and goat anti-rabbit secondary antibody were obtained from Cell Signaling (Beverly, MA, United States).

### *In vivo* Studies

#### Surgery and Drug Administration

Sixty rats were randomly assigned to five groups (groups A–E) with 12 rats in each group. This study was performed double-blind. Rats in groups D–E were pretreated with site-directed injection of the mast cell stabilizer cromolyn (200 μg/μl) into the hypothalamus, while rats in groups A–C were pretreated with 0.9% NaCl in the hypothalamus. After 30 min, rats in groups B to E were given intraperitoneal injection of LPS (1 mg/kg) while rats in group A were injected with 0.9% NaCl intraperitoneally. Rats in groups B and D were sacrificed 30 min after LPS injection, while rats in groups A, C, and E were sacrificed 24 h after LPS injection.

Mast cells are plentiful in hypothalamus. Therefore mast cell stabilizer cromolyn was centrally site-injected into the ipsilateral hypothalamus to determine whether mast cells are involved in LPS-induced neuroinflammation. As described in our previous report (Dong et al., 2017), the rats were anaesthetized by 50 mg/kg of pentobarbital sodium given intraperitoneally, then placed in a stereotaxic apparatus (Stoelting Instruments, United States). Guide cannulas (Plastic One) were inserted into the right hypothalamus of rats at 1.80 mm lateral and 1.90 mm posterior from Bregma, with a depth of 8 mm and at a 10° angle. After implantation, the rats were given 14 days to recover, with daily handling to check on the guide cannula. For the experiments involved, 1 μl of 200 μg/μl cromolyn (200 μg) or 1 μl of 0.9% NaCl was injected directly into the ipsilateral hypothalamus through the implanted guide cannulas. These rats were kept in their cages for 30 min without other restraint. Then, the rats were injected intraperitoneally with either LPS or 0.9% NaCl (control group). After drug administration, the rats were sacrificed and their brains were collected for morphological (*n* = 6) and biochemical (*n* = 6) analyses.

To evaluate the effects of LPS on microglia activation in mast cell-deficient mice, 12 Kit^W-sh/W-sh^ and 12 wild-type (WT) mice were each divided into two equal groups, of which one received intraperitoneal LPS (*n* = 6) and the other received 0.9% NaCl (*n* = 6).

#### Mast Cell Staining and Counting

Rats were anesthetized with chloral hydrate, then perfused with 0.9% NaCl followed by 4% cold paraformaldehyde in 0.1 M phosphate-buffered saline (PBS) at pH 7.4. The brains were dissected out and maintained overnight in 4% paraformaldehyde, then cryopreserved in PBS containing 30% sucrose before being stored at -70°C until use. Free-floating sections encompassing the entire brain were prepared using a cryostat, then stained with 0.05% toluidine blue and counted as previously described (Dong et al., 2017). Briefly, a 1% stock solution of toluidine blue in 70% ethanol was dissolved in 0.5% NaCl (pH 2.2–2.3). The slides were immersed in this staining solution for 30 min, then washed twice with distilled water and dehydrated using a series of increasing concentrations of ethanol, and finally immersed in butyl acetate ester. Cover slips were applied using Eukitt^®^ mounting medium and the slides were allowed to dry overnight.

The entire surface area of the ipsilateral and contralateral thalamus was scanned manually using a light microscope at 200× magnification. Mast cells were counted under double-blind conditions with the help of the Cell D software (Olympus) and expressed as the number of cells in the high power field. Mast cells were considered degranulated based on the following criteria: loss of purple staining, fuzzy appearance, distorted shape, or multiple granules visible near the cell.

#### Immunohistochemical and Immunofluorescence Analysis

Manual immunochemical and immunofluorescence analyses of brain sections were performed as previously described (Dong et al., 2017). Brain sections of rat and mice were obtained by the method described above. Then, rat brain sections were processed for immunohistochemistry, and mouse brain sections were processed for immunofluorescence.

For immunochemistry analyses, rat brain tissue section (30 μm) were incubated for 1 h in 10% bovine serum albumin (BSA) with 0.3% Triton X-100 in 0.01 M phosphate-buffered saline, then overnight with primary antibodies at 4°C. The primary antibodies used in this experiment were rabbit anti-Iba1 (1:200) and mouse anti-tryptase (1:100). Tissue sections were washed and incubated in the following day with goat anti-mouse or anti-rabbit secondary antibodies for 1 h at room temperature. Immunostaining was visualized with 3, 3′- diaminobenzidine, after which sections were counterstained with hematoxylin. The slides were scanned using a Leika 2500 (Leica Microsystems, Wetzlar, Germany) at 200× magnification.

For immunofluorescence analysis, mouse brain sections were incubated overnight with rabbit anti-Iba1 monoclonal antibody (1:200) in blocking solution at 4°C. Tissue sections were washed three times with PBS, then incubated with PE-conjugated goat anti-rabbit secondary antibody (1:200) at 37°C for 1 h. Cell nuclei were stained with DAPI. Fluorescent images were acquired using a confocal microscope (Leica, Frankfurt, Germany). Counts of Iba-positive cells were determined with the help of the Cell D software (Olympus) and expressed as the number of cells in the high power field.

### Behavioral Analysis

#### Y Maze

The Y maze was previously described (Lu et al., 2015). To adapt to the environment, one rat was placed at the end of a randomly-chosen arm and allowed to move for 3 min without stimulation. Then the test started, with foot stimulation given until the rat reached the illuminated arm (safe region). During each test, we used a randomized method to vary the orientation of the safe and stimulation regions. The test was considered correct (learned) if it reached the safe region within 10 s, and nine correct responses out of 10 consecutive foot stimulations (9/10 standard) were required to consider the rat as having reached the learning criterion. All rats reached the learning criterion in the present study. Learning ability was defined as the total number of stimulations needed to reach the criterion during training.

#### Trace Fear Conditioning (TFC)

Hippocampal–dependent memory in rats was assessed as previously described (Feng et al., 2013). Twenty-four rats were trained to associate an unconditional stimulus (foot shock) and a conditional stimulus (tone) with environment. The training model as followed: shock duration of 2 s, and shock intensity of 0.8 mA; a tone duration of 20 s, and sound level of 80 dB (Sun et al., 2015). Cromolyn was given immediately after the fear conditioning paradigm, and IP LPS injection was performed 30 min later. The training was comprised of an initial exploratory phase (100 s), followed by two trials with a 100 s interval. The trials included a 20 s auditory cue (80 dB, 5 kHz), followed by a 2 s foot shock (0.8 mA). Rats anticipate the shock by “freezing,” which is defined as the absence of all movement except respiration; this defensive posture reflects learned fear. When placed in the same context on a subsequent occasion, the learned fear is recalled and the degree of learning and recall can be determined from the extent of freezing. Contextual memory of the learned fear was assessed 1 day after the LPS injection, and freezing behavior in the absence of the tone and shock was automatically scored by video tracking software (Xeye Fcs, Beijing MacroAmbition S&T Development Co., Ltd., Beijing, China) over the course of 300 s. Freezing scores for each subject were expressed as a percentage of the total testing time.

### Flow Cytometry Analysis

Flow cytometry was employed to determine microglial activation in mice and receptor expression in rat microglia. As previously described (Dong et al., 2017), the dissociated cells from ipsilateral hypothalamus tissues were incubated with appropriate primary antibody overnight at 4°C, then incubated with 1 μg/ml of FITC-conjugated goat anti-rabbit secondary antibody for 1 h at 37°C. FACSCalibur flow cytometer (BD Biosciences, United States) was used to analyze the cells. For analysis of rat ipsilateral hypothalamus tissues, the primary antibodies were rabbit anti-H_1_R, anti-H_2_R, anti-H_3_R, anti-H_4_R, anti-PAR2, and anti-TLR4, or normal rabbit IgG, while FITC-conjugated goat anti-rabbit was used as the secondary antibody with PE-conjugated mouse anti-OX-42 antibody or isotype control (1:200). For analysis of mouse tissues, the primary antibody was rabbit anti-Iba1 or normal rabbit IgG, and the secondary antibody was FITC-conjugated goat anti-rabbit.

### *In vitro* Studies

#### P815 Cell Culture

The P815, mast cells line derived from mouse tumor cells was kindly provided by Fu Ning, PhD, Department of Immunology, Southern Medical University. As previously described (Zhang X. et al., 2016), the cells were incubated with DMEM medium containing 10% FCS at 37°C in a humidified atmosphere of 5% CO_2_/95% air. For assays, P815 cells (1 × 10^6^ cells) were treated with or without LPS (1 μg/ml) for 12, 24, 48, and 72 h.

#### Microglia-Enriched Cultures

Mice primary microglia were prepared as previously described with slight modifications (Dong et al., 2014). Briefly, brain tissues of postnatal (P1–P2) BABL/c mice were grinded; the resulting cells were cultured in poly-D-lysine precoated cell culture flasks with DMEM containing 10% fetal calf serum, 100 U/ml penicillin, and 100 mg/ml streptomycin. Cultures were maintained at 37°C in a humidified atmosphere of 5% CO_2_/95% air. After reaching a confluent monolayer of glial cells (10–14 days), microglia were separated from astrocytes by shaking for 5 h at 100 r.p.m., then replanted on 24-well culture plates at a density of 10^5^ cells/cm^2^. The enriched microglia were > 98% pure as determined by expression of Iba1.

#### Co-culture of P815 Cells and Microglia

As previously described (Zhang X. et al., 2016), P815 cells (1 × 10^6^ cells) were treated with cromolyn for 30 min, then cells were stimulated with LPS (1 μg/ml) for 12, 24, 48, and 72 h. Primary microglia (1 × 10^6^ cells) were treated with conditioned medium (CM) from P815 cells with or without LPS treatment for the given time periods. The conditioned cells were further incubated for 6, 12, and 24 h. In addition, we also stimulated microglia with cromolyn (10 μg/ml), LPS (1 μg/ml), or LPS and cromolyn together.

### Cytokines Assay

The expression of TNF-α and IL-6 in rat ipsilateral and contralateral hypothalamus or ipsilateral and contralateral cerebral cortex tissue extracts were quantified with a commercial ELISA kit from R&D Systems (Minneapolis, MN, United States). Histamine and mast cells tryptase contents in the supernatant of mast cells were tested with a detection ELISA kit from Fitzgerald (Birmingham, United Kingdom). The levels of selected cytokines (TNF-α, IL-6, IL-1β, INF-γ, RANTES, and IL-10) in the culture media were measured with a Milliplex kit (Merck & Millipore, United States) following the manufacturer’s instructions. All samples were run in duplicate.

### Western Blotting

Ipsilateral hypothalamus tissue extracts and primary microglia cells were collected and homogenized in lysis buffer. The cell lysate was used to assess protein expression by western blotting as previously described (Dong et al., 2017). The primary antibodies were: rabbit antibodies against JNK, phospho-JNK, p38, phosphop38, ERK, phospho-ERK, AKT, and phosphoAKT (1:1000). And the secondary antibody was goat-anti-rabbit (1:1000). Protein bands were detected with an enhanced chemiluminescence kit (Thermo Fisher Scientific, Waltham, MA, United States).

### Flow Cytometry Analysis

Microglia were pelleted by centrifugation at 450 g for 10 min, then fixed in 4% paraformaldehyde for 30 min. After washing, the cells were re-suspended in PBS. Cells were incubated overnight at 4°C with rabbit anti-H_1_R, anti-H_2_R, anti-H_3_R, anti-H_4_R, anti-PAR2, and anti-TLR4 antibodies or normal rabbit IgG. Subsequently, they were incubated at 37°C for 1 h, followed by 1 μg/ml of FITC-conjugated goat anti-rabbit secondary antibody or isotype control (1:200). Finally, the cells were re-suspended in PBS. FACSCalibur flow cytometer (BD Biosciences, United States) was used to analyze the cells.

### Statistical Analysis

All values are expressed as means ± SD. Significant differences (*P* < 0.05) between treatments and control were determined by one-way ANOVA followed by the *post hoc* least significant difference test.

## Results

### Cromolyn Alleviated LPS-Induced Mast Cell Activation and Memory Impairment

**FIGURE 1:**
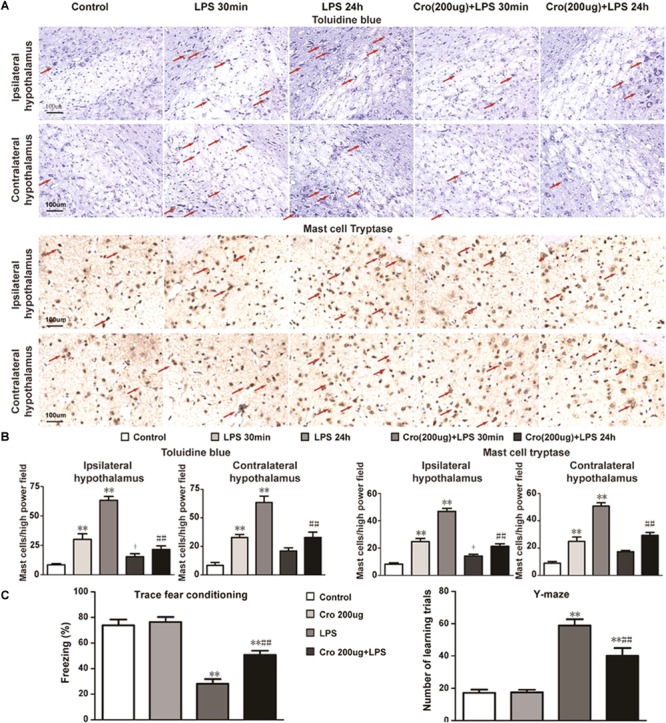
Cromolyn repressed LPS-induced mast cell activation in rat hypothalamus and memory impairment. **(A)** Rat brain mast cells were stained with toluidine blue and Mast Cell Tryptase. Scale bar: 100 μm. **(B)** Quantification of activated mast cells in rat ipsilateral and contralateral hypothalamus. ^∗∗^*P* < 0.01 vs. Control group. ^+^*P* < 0.05 vs. LPS 30 min group, ^##^*P* < 0.01 vs. LPS 24 h group. **(C)** Context fear response, as measured by freezing behavior, was determined in the rats. The Y-maze test was performed after TFC in the rats. ^∗∗^*P* < 0.01 vs. Control group. ^##^*P* < 0.01 vs. LPS 24 h group. The data are presented as the mean ± SD (*n* = 6).

To evaluate whether brain mast cells are involved in LPS-induced neuroinflammation, we determined the activation of brain mast cells in the hypothalamus at 30 min or 24 h after LPS injection. Brain mast cells were quantified in tissue sections stained with toluidine blue (TB) and mast cell tryptase (Figure 1A). As shown in Figure 1B, intraperitoneal injection of 1 mg/kg LPS 30 min or 24 h induced a significant increase in the number of activated mast cells in both the ipsilateral and contralateral hypothalamus as compared with that in the control group. Treatment with mast cell stabilizer cromolyn (200 μg) repressed the mast cell activation induced by LPS IP injection.

Rats were pretreatment of cromolyn (200 μg) 30 min before LPS administration to determine the effect of mast cells on LPS-induced memory impairment. Contextual assessment and Y-maze test were used to assess rats’ cognitive function after LPS treatment for 1 day. As shown in Figure 1C, the rats exposed to LPS alone exhibited a significant reduction in cognitive function compared to those given only saline. Pre-treatment with cromolyn significantly promoted freezing behavior and the number of learning trials, suggesting cromolyn alleviates LPS-induced memory dysfunction. Together, these results indicate that mast cells play a role in memory impairment induced by LPS and cromolyn can limit the adverse cognitive outcomes caused by endotoxemia.

### Stabilization of Mast Cell Inhibited LPS-Induced Microglia Activation in Hypothalamus

**FIGURE 2:**
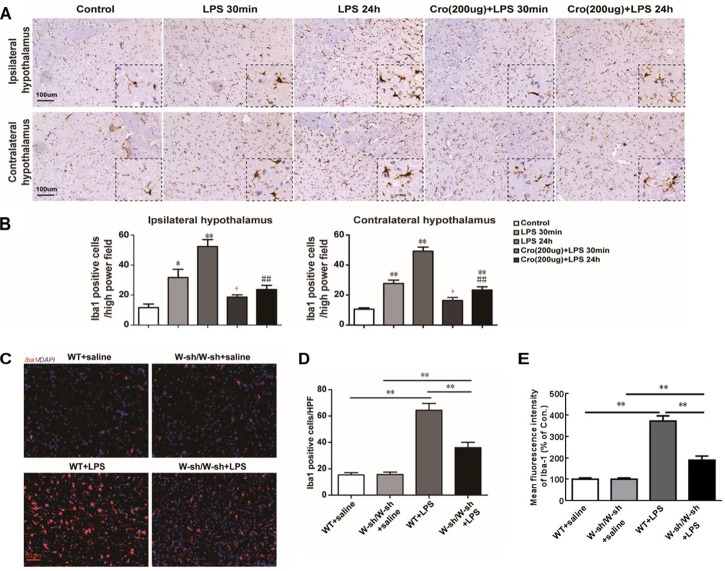
Stabilization of mast cell inhibited LPS-induced microglia activation in hypothalamus. **(A,B)** Immunohistochemical analysis was used to detect Iba1, markers of microglia, expression in rat hypothalamus. The activated microglia cells had larger cell body, poorly ramified short and thick processes. ^∗^*P* < 0.05, ^∗∗^*P* < 0.01 vs. Control group. ^+^*P* < 0.05 vs. LPS 30 min group, ^##^*P* < 0.01 vs. LPS 24 h group. **(C)** The effect of LPS on microglia activation was decreased in mast cell-deficient Kit^W-sh/W-sh^ mice. Immunofluorescence was used to detect Iba1 expression in mice hypothalamus. **(D)** Quantification of Iba1 positive cells in the hypothalamus. **(E)** For flow cytometry analysis of the expression of Iba. ^∗∗^*P* < 0.01. Data are presented as the mean ± SD (*n* = 6). HPF, high power field.

The effects of brain mast cells on LPS-induced microglia activation were determined through immunostaining for Iba1, a marker for microglia. IP injection of LPS induced a large number of microglia activation in both ipsilateral and contralateral hypothalamus. The cell morphology changes of activated microglia are processes retraction and cell body enlarged. And activated microglia were showed by notable Iba1-IR positive. Pretreatment of Cromolyn (200 μg) significantly suppressed LPS-induced activation of microglia in both ipsilateral and contralateral hypothalamus (Figure 2A). Quantification of Iba1 positive cells in the hypothalamus was shown in Figure 2B. However, the effect of Cromolyn is partial inhibition, but not completely reverse. These results indicate that stabilizing mast cells partial suppress LPS-induced activation of hypothalamus microglia.

The mast cell-deficient Kit^W-sh/W-sh^ mice were used to further confirm the role of mast cells in LPS induced microglia activation. As shown in Figure 2C,D, IP injection of LPS into WT mice induced significant microglia activation in the hypothalamus 24 h later, but had less effect in the Kit^W-sh/W-sh^ mice. Further characterization by flow cytometry (Figure 2E) demonstrated that WT mice stimulated with LPS expressed Iba-1 at levels three times higher than the corresponding saline group. However, mast cell-deficient Kit^W-sh/W-sh^ mice treated with LPS had only two times greater Iba-1 expression than their control group, indicating indicating that mast cells contribute to LPS-induced activation of microglia.

### Stabilization of Mast Cell Inhibited LPS-Induced Proinflammatory Factors Production and MAPK Activation in Hypothalamus

**FIGURE 3:**
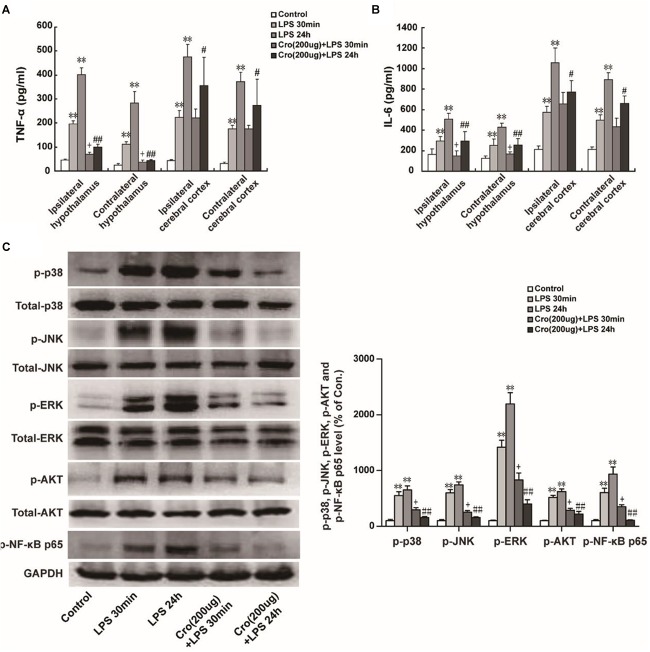
Stabilization of mast cell inhibited LPS-induced proinflammatory factors production and MAPK activation in hypothalamus. IP injection of LPS (1 mg/kg) for 30 min or 24 h significantly increased TNF-α **(A)** and IL-6 **(B)** expression in ipsilateral and contralateral hypothalamus or ipsilateral and contralateral cerebral cortex in rats. Furthermore, Cromolyn (200 μg) could partial inhibit the effect of LPS. ^∗∗^*P* < 0.01 vs. Control group. ^+^*P* < 0.05 vs. LPS 30 min group,^#^*P* < 0.05, ^##^*P* < 0.01 vs. LPS 24 h group. **(C)** Protein expressions of p-p38, p-JNK, p-ERK, p-AKT, and p-NF-κB p65 in ipsilateral hypothalamus of rats were examined by Western blotting. ^∗∗^*P* < 0.01 vs. Control group. ^+^*P* < 0.05 vs. LPS 30 min group, ^##^*P* < 0.01 vs. LPS 24 h group. Data are presented as the mean ± SD (*n* = 6).

Excessive proinflammatory cytokines released from activated microglia is involved in microglia-mediated neuroinflammation. The proinflammatory cytokines IL-6 and TNF-α were detected in present study. We found that IP injection of LPS (1 mg/kg) significantly promoted TNF-α release in ipsilateral and contralateral hypothalamus. IP injection of LPS also upregulated IL-6 and TNF-α content in ipsilateral cerebral cortex and contralateral cerebral cortex. Cromolyn (200 μg) was able to alleviate TNF-α and IL-6 levels in the ipsilateral and contralateral hypothalamus induced by LPS injection of 30 min or 24 h (Figure 3A,B). Site-directed injection of cromolyn in the hypothalamus also inhibited the production of TNF-α and IL-6 in cerebral cortex at 24 h after LPS injection, but it is not statistically significant on IL-6 and TNF-α levels in cerebral cortex induced by LPS injection of 30 min. These results indicated that stabilizing mast cells prevents LPS-induced proinflammatory cytokines release.

Mitogen-activated protein kinases (MAPK) and NF-κB are the predominant signaling transduction pathways responsible for the synthesis and production of proinflammatory mediators in LPS-induced neuroinflammation and microglia activation (Akundi et al., 2005; Ciallella et al., 2005). Phosphorylation of AKT is a downstream target of PI3K activation and therefore is a proxy for activation of the PI3K pathway (Desai and Thurmond, 2011). We investigated whether cromolyn could affect LPS-induced phosphorylation of MAPK and AKT. As shown in Figure 3C, IP injection of LPS for 30 min or 24 h induced MAPK, AKT, and NF-κB p65 phosphorylation. Cromolyn (200 μg) was given 30 min before LPS treatment partially inhibited LPS-induced MAPK, AKT, and NF-κB p65 phosphorylation. These results indicate that stabilization of mast cells suppress LPS-induced MAPK, AKT, and NF-κB signaling pathway activation.

### LPS Changed Receptor Expression in Hypothalamus Microglia

**FIGURE 4:**
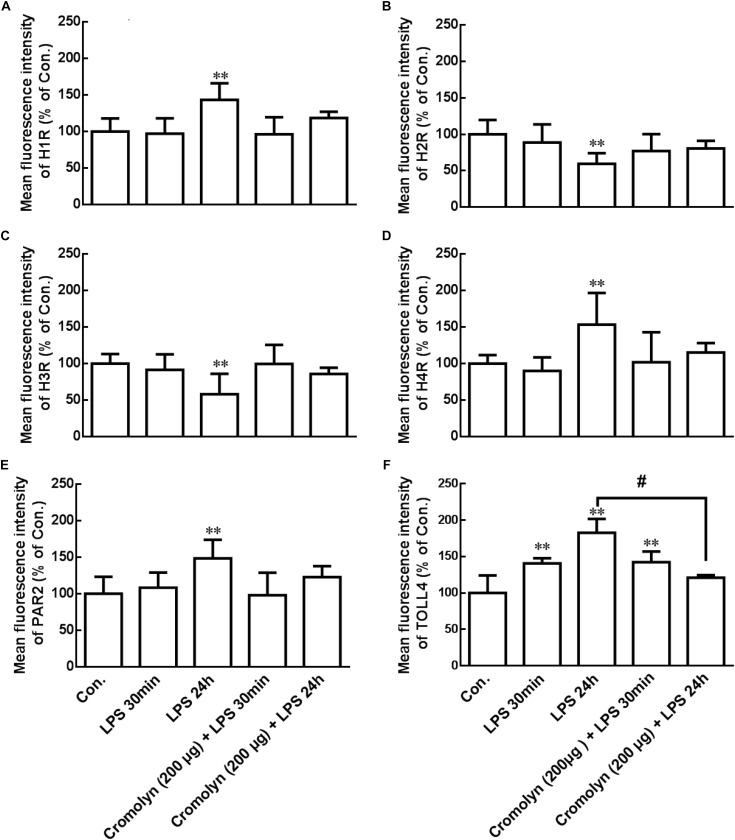
LPS induced receptor change in microglia of hypothalamus. Flow cytometry analysis was used to determine the receptor expression of H_1_R, H_2_R, H_3_R, H_4_R, PAR2, and TLR4 in the rat ipsilateral hypothalamus microglia. Mast cell stabilizer cromolyn inhibits LPS-induced TLR4 increase on microglia in rat hypothalamus. ^∗∗^*P* < 0.01 vs. Control group. ^#^*P* < 0.05 vs. LPS 24 h group. Data are presented as the mean ± SD (*n* = 6).

Degranulated mast cells can release tryptase and histamine, which induced microglial activation and inflammatory cytokines release (Zhang S. et al., 2012; Dong et al., 2014). Flow cytometry analysis was used to explore whether LPS-induced activated mast cells can change some receptor proteins expression in microglia. As shown in the Figure 4, the expressions of H_1_R, H_4_R, PAR2, and TLR4 were upregulation, but H_2_R and H_3_R expressions were deregulation in the ipsilateral hypothalamus microglia after IP injection of LPS for 24 h. Pretreatment of cromolyn (200 μg) 30 min before LPS administration inhibited LPS-induced TLR4 upregulation. However, cromolyn only had a tendency to inhibit the effect of LPS on other receptor protein expression in microglia, but it has no statistical significance. These results indicate that LPS-induced mast cells activation change some receptor expression in hypothalamus microglia, and mast cell stabilizer cromolyn had a tendency to inhibit the effect of LPS.

### Cromolyn Inhibited LPS-Induced P815 Cells Degranulation

**FIGURE 5:**
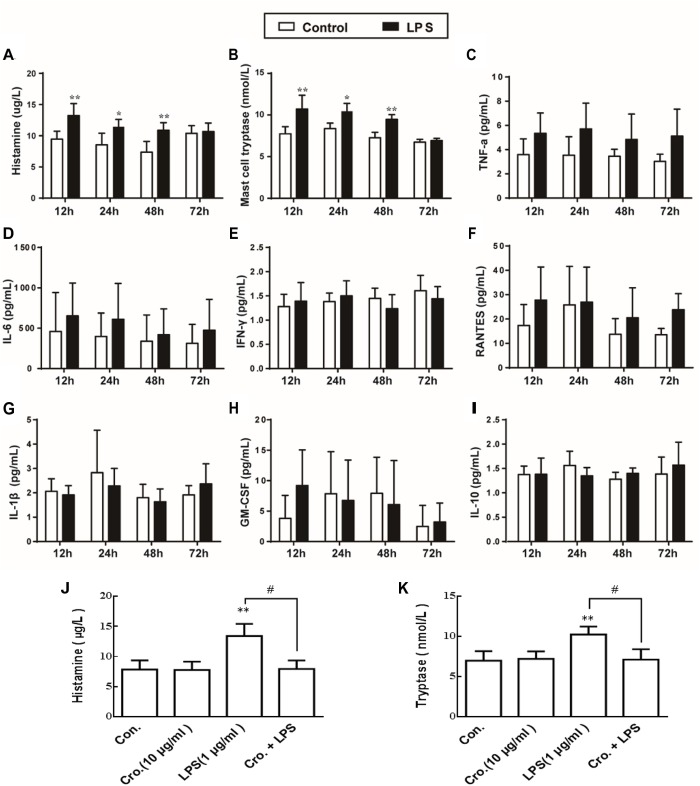
Cromolyn inhibited LPS-induced P815 cells degranulation. P815 cells were treated with LPS for 12, 24, and 48 h. ELISA was used to detect the expression of **(A)** histamine, **(B)** tryptase, **(C)** TNF-α, **(D)** IL-6, **(E)** INF-γ, **(F)** RANTES, **(G)** IL-1β, **(H)** GM-CSF, and **(I)** IL-10 in the medium. Cromolyn inhibited LPS-induced histamine **(J)** and tryptase **(K)** release from P815 cells. ^∗^*P* < 0.05, ^∗∗^*P* < 0.01 vs. Control group. ^#^*P* < 0.05 vs. LPS group. The data are presented as the mean ± SD (*n* = 4).

To observe the mast cells activation and the effect of LPS on mediator release, we quantified the expression levels of histamine and mast cell tryptase in the supernatant after stimulation with LPS based on previously described method (Zhang X. et al., 2016). As shown in Figure 5A,B, LPS (1 μg/ml) stimulation of P815 cells for 12, 24, and 48 h all increased the level of histamine and tryptase, indicating that degranulation was induced. We also quantified the secretion of a number of cytokines (TNF-α, IL-6, IFN-γ, RANTES, IL-1β, GM-CSF, and IL-10). In contrast to histamine and mast cell tryptase released, stimulation of P815 with LPS for 48 h had no effect on the levels of secreted cytokines (Figure 5C–I). Treatment with cromolyn (10 μg/ml) alone did not induce histamine and tryptase release from P815, but did inhibit the LPS-induced histamine and tryptase release from P815 cells (Figure 5J,K). These results indicate that LPS treatment (12–48 h) can induce P815 cells degranulation, and this process is inhibited by the mast cell stabilizer cromolyn.

### Cromolyn Inhibited Activated P815 Cell-Induced Pro-inflammatory Cytokines Production From Microglia

**FIGURE 6:**
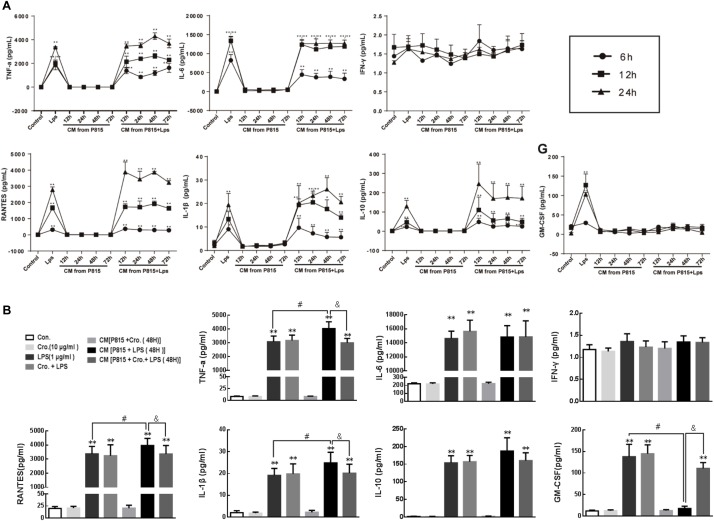
Cromolyn inhibited activated P815 cell-induced pro-inflammatory cytokines production from microglia. **(A)** Primary microglia was treated with CM from P815 cells with or without LPS stimulation for 12, 24, 48, and 72 h for different incubation times (6, 12, and 24 h). ELISA was used to detect the expression of TNF-α, IL-6, INF-γ, RANTES, IL-1β, IL-10, and GM-CSF. **(B)** TNF-α, RANTES, and IL-1β levels in microglia stimulated by CM from P815 with LPS (48 h) were higher than that stimulated by LPS alone at longer time points (24 h), which was inhibited by mast cell stabilizer cromolyn (10 μg/ml). ^∗∗^*P* < 0.01 vs. Control group. ^#^*P* < 0.05 vs. LPS group. ^&^*P* < 0.05 vs. CM from P815 with LPS (48 h) group. The data are presented as the mean ± SD (*n* = 4).

Conditioned medium (CM) from P815 cells given different treatment was used to explore the effects of activated mast cells on the activation of primary microglia. Microglia were incubated with CM for an additional 6, 12, and 24 h. Cytokine levels (TNF-α, IL-6, IFN-γ, RANTES, IL-1β, GM-CSF, and IL-10) in the supernatant were quantified to estimate the levels of microglia activation. Compared with control group, CM from P815 cells treated with LPS (1 μg/ml) evoked inflammation-related cytokines TNF-α, IL-6, RANTES, IL-1β, and IL-10 production from microglia (Figure 6A).

However, residual LPS in the CM will continue to stimulate microglia; therefore, we cannot conclusively determine the effect of this CM on microglia activation. To address this issue, cytokines levels in the supernatant of microglia stimulated by LPS alone and in the supernatant of microglia stimulated by CM from P815 cells with LPS were compared. As shown in the Figure 6B, the levels of pro-inflammatory cytokines TNF-α, RANTES, and IL-1β were significantly upregulated for microglia stimulated by CM from LPS-induced P815 (48 h) than that stimulated by LPS alone at longer timepoints (24 h). This process was inhibited by treatment with the mast cell stabilizer cromolyn (10 μg/ml). We also found that the level of GM-CSF released from microglia was significantly inhibited by CM from P815 with LPS as compared with that by LPS alone, which effect was partially reversed by cromolyn. However, there was no significant difference observed in between the level of anti-inflammatory cytokine IL-10 in microglia stimulated by CM from P815 with LPS and that stimulated by LPS alone. We also found that cromolyn alone had no effect on cytokine production from microglia with or without LPS. Therefore, the inhibitory effect of cromolyn on microglia activation relies on reducing mast cell activation.

### MAPK Signaling Pathways Were Involved in the Mast Cells-Induced Microglia Activation

**FIGURE 7:**
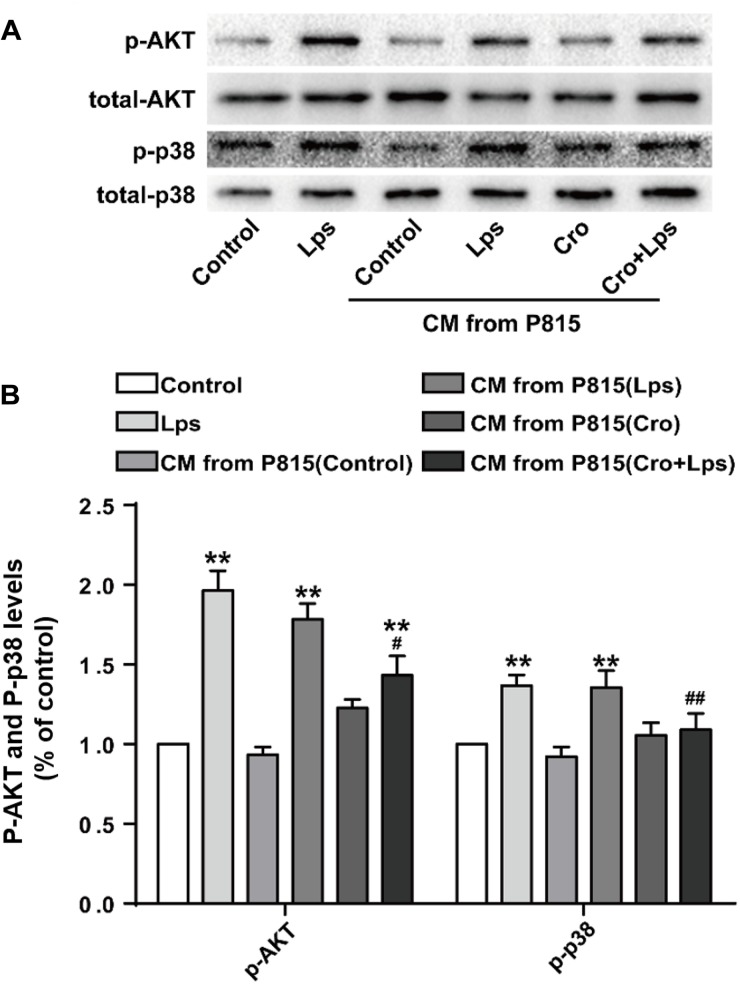
MAPK signaling pathways were involved in the mast cells-induced microglia activation. **(A)** The expression of p-AKT and p-p38 were detected by Western blotting. **(B)** Levels of p-AKT and p-p38 were quantified and normalized to GAPDH levels. Each value was expressed relative to the control, which was set to 1. ^∗∗^*P* < 0.01 vs. Medium group. ^#^*P* < 0.05, ^##^*P* < 0.01 vs. CM from P815 with LPS (48 h) group. The data are presented as the mean ± SD (*n* = 4).

A previous *in vivo* study identified the MAPK and AKT signaling pathways as important in mast cells-induced microglia activation; we therefore validated the involvement of these signaling pathways *in vitro.* Treatment of microglia with CM from LPS-stimulated P815 cells resulted in sharp upregulation of phosphorylation of AKT and P38, this result was ameliorated by pretreatment with cromolyn (10 μg/ml) (Figure 7A,B). These changes were consistent with *in vivo* study, and support that MAPK signaling may be involved in the activation of microglia by mast cells.

### Cromolyn Inhibited Activated P815 Cell-Induced H_1_R, H_4_R, and TLR4 Increase in Microglia

*In vivo*, we found that LPS induced receptor protein expression change in microglia of the hypothalamus. Mast cell stabilizer cromolyn, however, can inhibit the effect of LPS. To further support this, the effect of LPS on receptor protein expression change in microglia was examined *in vitro* by flow cytometry.

**FIGURE 8:**
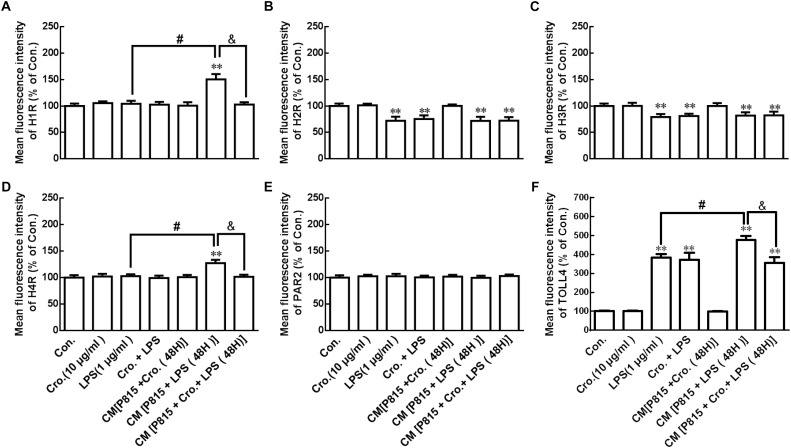
Cromolyn inhibited activated P815 cell -induced H_1_R, H_4_R, and TLR4 increase in microglia. Flow cytometry analysis was used to determine the receptor expression of H_1_R, H_2_R, H_3_R, H_4_R, PAR2, and TLR4 in microglia. ^∗∗^*P* < 0.01 vs. Control group. ^#^*P* < 0.05 vs. LPS group. ^&^*P* < 0.05 vs. CM from P815 with LPS (48 h) group. The data are presented as the mean ± SD (*n* = 4).

P815 cells were stimulated by treatment with LPS (1 μg/ml) for 24 h. As shown in Figure 8, this stimulation downregulated H_2_R and H_3_R, and upregulated TLR4 in microglia. Treatment with cromolyn (10 μg/ml) alone did not affect receptor protein expression in microglia with or without the stimulation of LPS, but did inhibit the increased expression of H_1_R, H_4_R, and TLR4 in microglia stimulated by CM from P815 with LPS (48 h) These results suggest that LPS-induced P815 cell activation can stimulate H_1_R, H_4_R, and TLR4 upregulation in microglia and that inhibitory effect of cromolyn acts only through mast cells.

## Discussion

One of the most important revelations in neuroscience research is the realization of communication between the immune system and CNS. Proinflammatory cytokines released by microglia (the brain’s resident macrophages), play a key role in this communication, and also have fundamental roles in neurodegenerative diseases. Microglia also responds to mediators released from other immune cells, such as mast cells (Zhang S. et al., 2012; Dong et al., 2014). We have previously found that histamine, tryptase and mast cell degranulation can all induce microglia activation. However, the role of mast cells in LPS-induced microglia activation, neuroinflammation and cognitive impairment has not been clarified. In this paper, we report that the stabilization of mast cells inhibits LPS-induced microglia activation and neuroinflammation and also alleviates LPS-induced cognitive dysfunction.

Neuroinflammation is an underlying pathological component in a wide range of neurodegenerative diseases, such as Parkinson’s disease, Alzheimer’s disease and multiple sclerosis (Skaper et al., 2017). In treating neuroinflammation, considerable efforts has been directed to restraining the inflammatory cascade of peripheral phagocyte and neutrophil infiltration and glia activation; less focus has been placed on the ability of resident brain cells, such as mast cells, to initiate an immediate host response in the meninges and cerebral parenchyma (Silver and Curley, 2013).

Microglia are regarded as the tissue-specific macrophages of the brain. Microgliosis is an important element of brain’s response to inflammation, which has an increased number of microglia (Ransohoff, 2007). In previous studies, we found that mast cell tryptase and histamine can stimulate microglia activation through the PAR2, H_1_R, and H_4_R (Zhang S. et al., 2012; Dong et al., 2014), and that mast cell degranulation induced by compound 48/80 can promote microglia activation (Dong et al., 2017). Thus, we hypothesized that mast cells may play a key role in neuroinflammation. In the present study, we found that IP injection of LPS for 30 min or 24 h induced activation of mast cells and microglia in both the ipsilateral and contralateral hypothalamus. Coincident with the change in microglia activation in the hypothalamus, we also observed an increase in the proinflammatory cytokines TNF-α and IL-6 contents of the hypothalamus and cortex, which may have been released from activated microglia. Levels of these cytokines were higher in the cortex, suggesting that LPS induces more production of proinflammatory cytokines in the cortex than in the hypothalamus.

Mast cells are found on the brain side of the BBB. They lie in close proximity to the basal side of blood vessel walls (Khalil et al., 2007), and they act not only as first responders in harmful situations but also as environmental “sensors” to communicate with glia, the extracellular matrix, and even neurons (Lindsberg et al., 2010). Activated brain mast cells can promote BBB breakdown and neutrophil infiltration, resulting in neuroinflammation that contributes to postoperative cognitive dysfunction. Therefore, inhibition of mast cell activation should be neuroprotective. In the present study, inhibiting mast cell degranulation by the mast cell stabilizer cromolyn limited microglia activation and release of TNF-α and IL-6, and alleviated LPS-induced cognitive impairment. Specifically, site-directed injection of cromolyn into the hypothalamus inhibited the production of TNF-α and IL-6 induced by LPS in both hypothalamus and cerebral cortex. However, the inhibitory effect on cortex cytokine production occurred only at 24 h after LPS injection; no significant effect was observed at 30 min after injection. This may be because the number of mast cells in the cerebral cortex is much less than that in the hypothalamus, so the effects of cromolyn are slowed in the cortex.

The mast cell deficient Kit^W-sh/W-sh^ mice were used to further demonstrate the role of mast cells in LPS-induced neuroinflammation. We found that LPS IP injection induced significantly microglia activation in WT mice, while LPS had less effect on microglia in Kit^W-sh/W-sh^ mice, indicating that activated mast cells, as “first responders,” can evoke, expand and prolong immune responses (Silver and Curley, 2013).

The factors from brain mast cells that responsible for the over activation of microglia have not been illuminated to date. Mast cells are characterized by a rapid release a large number of chemokines via degranulation (Feuser et al., 2012). Mast cell secretory mediators released in the CNS change the functions of T cell (Jutel et al., 2001), vascular elements (Esposito et al., 2002), and neuron (Koszegi et al., 2006). These cytokines further induce microglia activation (Chakraborty et al., 2010; Skuljec et al., 2011). We have found *in vitro* that tryptase released from mast cells induced microglia activation through PAR2-MAPK-NF-κB signaling pathways (Zhang S. et al., 2012), and histamine induced microglia activation via the H_1_R and H_4_R-MAPK and PI3K/AKT- NF-κB signaling pathways (Dong et al., 2014). *In vivo*, we found that site-directed injection of the mast cell degranulator Compound 48/80 in the hypothalamus induced microglia activation and increased microglial expression of H_1_R, H_4_R, PAR2, and TLR4 (Dong et al., 2017). Here, we demonstrate that IP injection of LPS also significantly increased the expression of H_1_R, H_4_R, PAR2, and TLR4 in microglia after 24 h. After LPS-induced degranulation of mast cell, the released mediators combine with these receptors on microglia to induce microglial activation, followed by activation of the MAPK and AKT-NF-κB signaling pathways. These contribute to exacerbate neuroinflammation-related disease (Skaper et al., 2014). Notably, pretreatment with cromolyn (200 μg) 30 min before LPS administration inhibited LPS-induced TLR4 upregulation. Cromolyn treatment also tended to inhibit the effects of LPS on microglial expression of other receptor proteins, but the results were not statistically significance due to high SD values. Nonetheless, we cannot completely deny the inhibitory effect of cromolyn on LPS-induced receptor protein expression.

*In vitro*, we found that 1 μg/ml LPS stimulated P815 mast cells to release histamine and tryptase, but had no effect on mast cell production of TNF-α, IL-6, IFN-γ, RANTES, IL-1β, GM-CSF, or IL-10. This suggests that TNF-α is not actually released from mast cells, and therefore mast cells have no role in LPS-induced inflammation mediated by TNF-α. Furthermore, the mast cell stabilizer cromolyn inhibits LPS-induced mast cell degranulation.

We also found that in the absence of mast cells, cromolyn had no effect on the production of cytokines by microglia, with or without LPS stimulation. In contrast, the conditioned media (CM) from LPS-induced P815 mast cells could induce microglia to release TNF-α, IL-6, RANTES, IL-1β, and IL-10. Notably, LPS remains present in the conditioned media in at least trace amounts, and can activate microglia directly. However, TNF-α, RANTES, and IL-1β levels 48 h after stimulation by CM from LPS-induced P815 cells were higher than in microglia stimulated by LPS alone (24 h). Furthermore, CM stimulation of microglia was inhibited by if the mast cells were also treated with the mast cell stabilizer cromolyn (10 μg/ml). We also found that the release of GM-CSF from microglia was significantly inhibited by CM from LPS-induced P815 cells comparing to that induced by direct LPS, and this release was partially inhibited by cromolyn. Histamine and tryptase released from LPS-induced P815 cells may have a role in inhibiting GM-CSF release from microglia; further research is needed to confirm this effect.

While most cytokines released by microglia activated by LPS are pro-inflammatory, LPS also induces release of the anti-inflammatory cytokine IL-10. Notably, we observed no significant difference in IL-10 levels in microglia stimulated by CM from LPS-induced P815 compared to those stimulated by LPS alone, suggesting that IL-10 release was solely induced by LPS. This implies that activated mast cells can only provoke the release of pro-inflammatory cytokines from microglia. These findings are also consistent with our previous report that released products of activated P815 cells changed microglial phenotypes toward M1/2b (Zhang X. et al., 2016). Therefore, mast cells may have a role in modulating the timing of cytokine release by microglia, thereby prolonging the inflammatory response.

In summary, the present study demonstrates that LPS can induce mast cell degranulation, which stimulates the production of inflammatory factors by activated microglia, and this process can be inhibited by the “mast cell stabilizer” cromolyn. These results imply that mast cell degranulation can aggravate LPS-induced neuroinflammation by evoking microglia activation, and the stabilization of mast cells can inhibit LPS-induced neuroinflammation and memory impairment. Investigating the role of mast cell activation in neuroinflammation is an important emerging research topic that needs to be explored in order to understand and effectively treat neuroinflammation-related diseases.

## Data Availability

All datasets generated for this study are included in the manuscript and/or the supplementary files.

## Author Contributions

SZ and HDi conceived and designed the study. HDo, XianZ, XiaoZ, and YW developed the methodology. HDo, YW, and XiaoZ acquired the data. SZ, HDo, and YQ analyzed and interpreted the data. HDo, SZ, and YW wrote, reviewed, and/or revised the manuscript. SZ supervised the study.

## Conflict of Interest Statement

The authors declare that the research was conducted in the absence of any commercial or financial relationships that could be construed as a potential conflict of interest.
